# A Lifetime Maximization Relay Selection Scheme in Wireless Body Area Networks

**DOI:** 10.3390/s17061267

**Published:** 2017-06-02

**Authors:** Yu Zhang, Bing Zhang, Shi Zhang

**Affiliations:** State Key Laboratory of Integrated Services Networks, Xidian University, Xi’an 710071, China; joquinzhang@163.com (Y.Z.); shzhang@mail.xidian.edu.cn (S.Z.)

**Keywords:** WBANs, lifetime, energy consumption, residual energy, relay selection, optimization, heuristic solution, IEEE 802.15.6

## Abstract

Network Lifetime is one of the most important metrics in Wireless Body Area Networks (WBANs). In this paper, a relay selection scheme is proposed under the topology constrains specified in the IEEE 802.15.6 standard to maximize the lifetime of WBANs through formulating and solving an optimization problem where relay selection of each node acts as optimization variable. Considering the diversity of the sensor nodes in WBANs, the optimization problem takes not only energy consumption rate but also energy difference among sensor nodes into account to improve the network lifetime performance. Since it is Non-deterministic Polynomial-hard (NP-hard) and intractable, a heuristic solution is then designed to rapidly address the optimization. The simulation results indicate that the proposed relay selection scheme has better performance in network lifetime compared with existing algorithms and that the heuristic solution has low time complexity with only a negligible performance degradation gap from optimal value. Furthermore, we also conduct simulations based on a general WBAN model to comprehensively illustrate the advantages of the proposed algorithm. At the end of the evaluation, we validate the feasibility of our proposed scheme via an implementation discussion.

## 1. Introduction

Wireless Body Area Networks (WBANs) have recently emerged as a subfield development of Wireless Sensor Networks (WSNs) and a promising technology in short-range communication area [1] to provide many important and useful applications in different domains such as vital sign monitoring, interactive gaming, and telemedicine [2,3,4]. In general, a WBAN consists of one coordinator and a set of sensor nodes that have to be very simple, tiny and harmless to the human body [5]. These sensor nodes are usually placed in the clothes or on the skin of a person, some even implanted into the body to monitor some certain parameters about human body. At the same time, the coordinator acts as a sink which collects all the information attained by the sensors and communicates it to the user or the remote server for further processing.

In order to provide satisfactory services in applications, network lifetime of WBANs ought to be highlighted as a crucial parameter [6,7]. As we know, sensor nodes deployed in WBANs are minimized in size, which means they are mostly supplied by non-renewable and energy-limited batteries. When one or more devices have to be implanted or worn by a person, enormous stress is caused by the battery replacement/recharging, which, in some cases, may require surgery. Furthermore, each node in WBANs has its unique function that can not be alternatively executed by other nodes. When one sensor runs out of power, WBANs will not perform well or even stop working. As a result, it is a necessity to prolong the lifetime of each sensor node in WBANs to relieve the stress of frequent recharging/replacement, especially the sensor node that may be exhausted first (The coordinator has sufficient energy compared to sensor nodes).

In IEEE 802.15.6 [8], which is specifically designed for WBAN communication, topology is specified commonly as a star topology and can be extended to a two-hop tree topology. This extension indicates that cooperative transmission of a sensor node through a relay is an alternative choice instead of direct one-hop transmission to the coordinator. The authors in [9] have proven that appropriately using cooperative transmission can effectively save energy and prolong the lifetime of sensor nodes that are relatively far away from the coordinator through mathematical analysis and numerical simulation. That is to say, rationally utilizing the combination of direct transmission and cooperative transmission to sensor nodes in WBANs can improve lifetime performance of the whole network. Hence, an appropriate relay selection is of great importance in cooperative transmission strategy to prolong the network lifetime of WBANs.

In this paper, a relay selection scheme called the Lifetime Maximization Relay Selection Scheme (short for LMRSS) is proposed to maximize the lifetime of WBANs through formulating and solving an optimization problem where relay selection of each node acts as the optimization variable under the topology constraints specified in the IEEE 802.15.6 standard. The proposed scheme not only regards the transmission depletion condition of each sensor node as relay selection criterion, but also considers the factor of energy difference among sensor nodes according to the fact that sensor nodes deployed on different locations of the human body have different sensing tasks and thus their node sizes and battery capacities may be diverse. Moreover, inferred from the fact that the network can function well only when all the nodes stay alive, in our formulated problem, the aim is to improve the minimum lifetime of sensor nodes within a WBAN, which provides maximum improvement of network lifetime.

In more designing detail of our relay selection scheme, we firstly derive that the lifetime of WBANs is a function of relay selection results. Then, regarding relay selection of each node as optimization variable, a network lifetime maximization problem that considers both energy consumption rate and residual energy is formulated with the target of maximizing the minimum lifetime of sensor nodes in the network. Considering that the optimization problem is an NP-hard binary integer problem and intractable, especially in the case where the number of the nodes in WBANs is large, a heuristic iterative solution is designed to rapidly solve the optimization problem with low time complexity. The performance of the proposed scheme is evaluated through numerical simulations. The results indicate that the proposed scheme has better performance in network lifetime compared with other existing methods in a human body scenario. Moreover, the results show that the heuristic solution can effectively reduce the time complexity with only a negligible performance degradation gap from the optimal value. In addition, to comprehensively illustrate the advantages of the proposed scheme, we also conduct simulations based on a more generalized WBAN model specified in the IEEE 802.15.6 standard. The simulation results demonstrate that the proposed scheme also excels in a more general application scenario. At the end of evaluation, we make a brief discussion on the implementation of our scheme in a real WBAN system to evaluate the feasibility of it.

The rest of this paper is organized as follows. Section 2 consists of related works. In Section 3, the system model considered in this paper is described. The optimization variables, optimization problem formulation and corresponding constraints are defined in Section 4. The heuristic solution is then introduced in Section 5. Simulation results, analyses and implementation discussion are presented in Section 6. Finally, we conclude the paper and briefly discuss the future works in Section 7.

## 2. Related Work

To extend network lifetime, there are already a number of routing protocols and relay selection algorithm for WSNs in the literature [10,11,12,13]. In [10], Youssef et al. proposed an energy-aware routing algorithm that uses a minimum number of hops for transmission of data. By varying the transmission distance, the interconnections between the nodes can be changed and different network topologies can be obtained. An energy balanced robust scheme based on swarm intelligence that chooses the next node based on node’s local information was suggested by Zhang and Shen [11]. This method balances load evenly among the nodes and is able to achieve longer lifetime. Another approach proposed in [12] reduces the total consumed energy based on two optimization objectives, i.e., path selection and bit allocation. Packets with the optimum size are relayed to the fusion node from sensor nodes in the best intermediate hops. In [13], a relay selection algorithm was proposed to formulate an optimization problem to maximize user data rates and minimize the total transmission power of the network.

However, porting these solutions from WSNs to WBANs is problematic due to the different network architectures and operating conditions. In WSNs, hundreds to thousands of sensor-nodes cover large areas, offering a considerable degree of redundancy, and use multi-hop communications. On the contrary, WBANs cover an area limited to the human body and offer no redundancy, only involving two hops. Data must be collected reliably under unique characteristics such as frequent varying channel conditions and transmission power restriction that is not presented in WSNs. In a word, an efficient relay selection algorithm should be designed specifically for WBANs.

In the literature [14,15,16,17], the authors proposed some relay-aided communication process for WBANs to enhance energy-efficiency. These works put an emphasis on the interaction between transmitter sensor and relay node with the consideration of link establishment procedure and overhead costs. However, how to select a suitable relay for a transmitter sensor is still not addressed in these works.

Routing protocols were also considered in the literature [18,19,20] to extend WBAN lifetime based on multi-hop topology. Nevertheless, these works violate the topology restriction in IEEE 802.15.6.

In [21], Elias proposed an energy-aware WBAN topology model, which optimizes the number and the location of relays to be deployed by an integer linear programming model to minimize the total energy cost of the network. A Ultra Wide Band(UWB)-based WBAN relay selection algorithm was proposed in [22]. The proposed algorithm utilizes an energy-efficient selection criterion to make relay selection for total power consumption minimization. Another cooperative transmission strategy for UWB-based WBAN was proposed in [23] using single-relay to improve the network lifetime. In the paper, an energy efficiency optimization problem is formulated on two practical on-body transmission scenarios. In [24], authors proposed a mechanism for WBANs to prolong network lifetime through formulating the network lifetime as a function of node transmission mode, cooperative node, transmission power and time slot, and maximizing the network lifetime subject to resource allocation constraints, then obtaining an optimal joint relay selection strategy. The authors in [25] presented a game-theoretic relay selection and power control method to investigate the problem of relay selection and power control with quality of service constraints in WBANs. The proposed method focused on energy efficiency, and its performances are examined in various scenarios. However, References [21,22,23,24,25] only concentrate on the energy consumption rate when selecting relays without considering sensor nodes’ other conditions like residual energy. When residual energy of each node is not the same, the lifetime enhancement of these algorithms will be degraded.

## 3. System Model

In this section, we first emphasize the conception of network lifetime of WBANs. Then, a brief description of the network model and the energy consumption model is presented based on which the proposed algorithm is implemented and analyzed.

### 3.1. Conception of Network Lifetime

As discussed before, a WBAN is very different from a WSN. In a WSN, network lifetime is generally specified as the time duration between the network initialization/restart to the point when the last sensor or the majority of sensor nodes die. However, in a WBAN, each sensor node is irreplaceable due to its unique function. The exhaustion of one sensor node may lead to network failure. As a consequence, network lifetime of a WBAN in this paper is denoted as the time duration between the initialization/restart of a WBAN to the point when the first sensor node in the network exhausts.

### 3.2. Network Model

**Figure 1:**
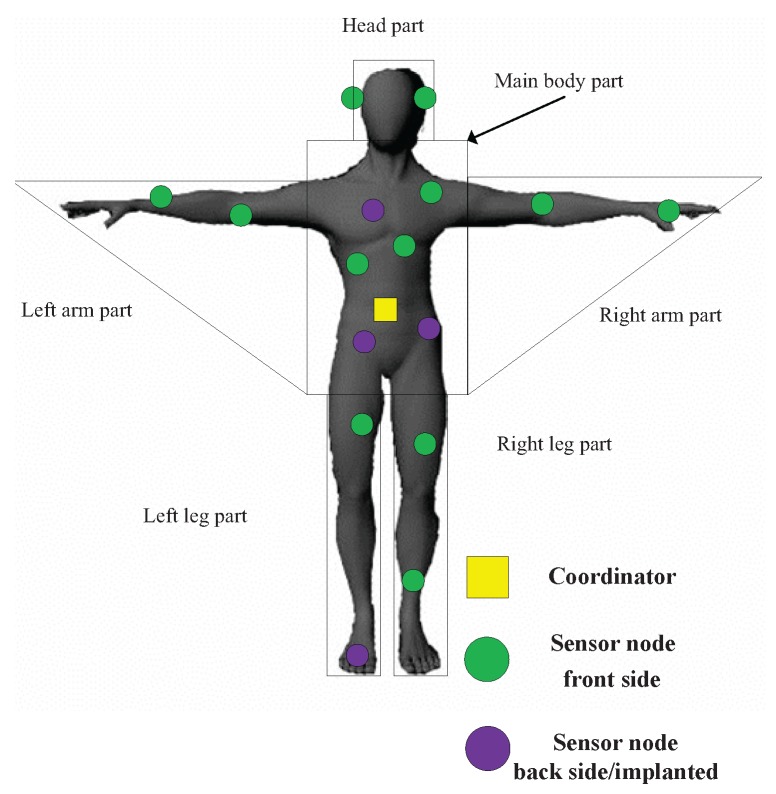
Network model.

In this paper, we consider a WBAN with one coordinator and *l* sensor nodes on a human body as shown in Figure 1. The coordinator is placed at the front side of abdomen while the sensor nodes are deployed in the different parts of the human body. Both direct transmission and cooperative transmission are allowed in the network. The sensor nodes deployed in the main body part can be selected as relay nodes because of their shorter distance from the coordinator. Relay nodes should not only transmit their own information to the coordinator, but also relay the information from some other nodes when selected as relay. In addition, relay nodes are allowed to use direct transmission only to comply with the IEEE 802.15.6 two-hop tree topology restriction. Only uplink data transmission from sensor nodes to the coordinator is considered due to application scenarios like health monitoring where most of the information transmitted are sensed data. We assume that the coordinator knows the network topology and the distance between each pair of nodes including itself.

A simple Time Division Multiple Access (TDMA) Media Access Control (MAC) is employed in this model to deal with multi-sensor transmission. Note that this MAC is a common used version of beacon-enabled superframes MAC specified in IEEE 802.15.6 (see [8] for more details). More specific, time is divided into superframes which has fixed length. A superframe has two parts: the active part and the inactive part. The active part consists of fixed-length time slots and each sensor node has one orthogonal time slot to send its sensed data to the coordinator without collision. If a sensor node is selected as a relay, more time slots are given to it. The number of the extra time slots for a certain relay is decided by the number of relayed sensors it has. Each sensor node transmits the sensed data to the receivers (either relay or the coordinator) in its dedicated slot, whereas relay nodes, if necessary, listen to their corresponding sensor nodes for data reception and transmit the relayed data together with their own data to the coordinator in their allocated slots. During the inactive part, nodes go to sleep mode. When a WBAN sets up or re-starts, the time slots allocation will be made by the coordinator depending on the relay selection results. Normal sensor nodes are allocated time slots first before relay nodes. In this paper, it is assumed that all nodes have enough sensed data to send during their allocated slots.

### 3.3. Energy Consumption Model

In WBANs, energy is consumed mainly on transmission, sensing and abnormal energy waste including idle listening, collision and overhearing. As described in the previous subsection, a TDMA MAC is implemented. Thus, it is considered that there is no energy consumed on abnormal energy waste. Moreover, the energy cost of sensing is so small that can be neglected when compared with transmission costs [26]. As a consequence, we focus on the energy consumed by transmission. We have chosen the transmission energy consumption model in [27,28], which is widely used in many WBAN related works. The energy cost to guarantee reliable transmission during transmitting and receiving are described in Equations (1) and (2) as follows:

(1) $$E_{tx}\left(k,d,n\right)=E_{TXelec}\cdot k+E_{amp}\cdot k\cdot d^{n},$$

(2) $$E_{rx}\left(k\right)=E_{RXelec}\cdot k.$$

The model takes $d^{n}$ as energy costs due to channel variation and the path loss in respect with distance *d* between sender and receiver. $E_{tx}$ represents the transmission energy, $E_{rx}$ the receiver energy, $E_{TXelec}$ and $E_{RXelec}$ the energy the radio dissipates to run the circuitry for the transmitter and receiver, respectively, and $E_{amp}$ is the energy for the transmitter amplifier. The specific values of these parameters are hardware dependent. In addition, *k* represents the number of bits sent for the transmission. *n* is pathloss coefficient related to shadow effect.

As introduced in Section 1, when making relay selection, the amount of energy in each node may not be always the same. Two conditions should be taken into consideration:

- A WBAN set up with sensor nodes with different battery capacities.
- A WBAN resumes from a restart when each sensor node has already depleted energy under different consumption rates.

The diversity of energy storage of each node in the network can bring obvious influence on the performance of WBANs. Hence, when making relay selection, the amount of energy in each node must be taken into consideration. As indicated from the reasons stated above, the energy difference among sensor nodes varies unpredictably. Therefore, according to the Central Limit Theorem and the indication, we adopt normal distribution to represent the energy storage condition of each sensor node. Meanwhile, we select an appropriate upper bound and lower bound for the energy storage values to avoid extreme large or small random values in normal distribution, which is impossible to appear in reality. In summary, the residual energy of the *i*th sensor node when making a relay selection in this paper follows truncated normal distribution which is represented as the following:

$$E_{i}∼N\left(\mu ,\sigma^{2}\right),\;\mu -\Delta \leq E_{i}\leq \mu +\Delta ,$$

where μ and Δ are standard values for the residual energy and maximum energy deviation from standard value in each node, respectively, while $\sigma^{2}$ stands for the energy difference degree among sensor nodes. Larger values of $\sigma^{2}$ reflects more residual inequality in the network.

## 4. Optimization Problem Formulation

In this section, the optimization variables, relay selection matrix and lifetime function are introduced first. Then, an optimization problem considering both energy consumption rate and residual energy of each sensor node, aiming to maximize the network lifetime of WBANs, is described in detail.

### 4.1. Variables and Matrix

In the formulation, $x_{i,j}$ is denoted as an optimization binary variable to represent relay selection results. $x_{i,j}$ = 1 represents the *i*th sensor node using cooperative transmission with the help of the *j*th sensor node, which is acting as a relay node; otherwise, $x_{i,j}$ = 0. It should be noticed that when *i* = *j*, $x_{i,i}$ (or $x_{j,j}$) = 1, this means that the *i*th (or *j*th) sensor node using direct transmission without any help from other nodes. Having known that there are *l* sensor nodes in the network, all of the binary variables $x_{i,j}$, i,j=1,2,⋯,l form a relay selection matrix X defined as follows:

$$X=\left[\begin{array}{ccccc} x_{1,1} & x_{1,2} & \cdots & x_{1,l-1} & x_{1,l}\\ x_{2,1} & x_{2,2} & \cdots & x_{2,l-1} & x_{2,l}\\ \vdots & \vdots & \ddots & \vdots & \vdots \\ x_{l-1,1} & x_{l-1,2} & \cdots & x_{l-1,l-1} & x_{l-1,l}\\ x_{l,1} & x_{l,2} & \cdots & x_{l,l-1} & x_{l,l} \end{array}\right].$$

In this matrix, the *i*th row represents the *i*th sensor node’s transmission strategy. That is to say, if $x_{i,j}=1,i\neq j$, the *i*th sensor node is to use cooperative transmission with relay of the *j*th sensor node; otherwise, if $x_{i,i}=1$, the *i*th sensor node is to use direct transmission. Due to the principle specified in IEEE 802.15.6 that each node can only transmit with the help of one relay node or by itself, $\sum_{j=1}^{l}x_{i,j}=1,\forall i=1,2,\cdots ,l$ always holds. In addition, in order to comply with the topology restriction, relay nodes only use direct transmission.

We also denote $d_{i,j},i\neq j$ as the distance between the *i*th sensor node and the *j*th sensor node. It should be noticed that when i=j, $d_{i,i}$ (or $d_{j,j}$) represents the distance between the *i*th(*j*th) sensor node and the coordinator.

### 4.2. Function and Problem Formulation

Defining $L_{i}$ as the lifetime of the *i*th sensor node, which stands for how many superframes the *i*th node can function well before it runs out, it can be mathematically represented as:

(3) $$L_{i}=\frac{E_{i}}{C_{i}},$$

where $C_{i}$ is defined as the *i*th sensor node’s energy consumption rate, which stands for the amount of energy consumed by the *i*th sensor node in one superframe. Using Equations (1) and (2), $C_{i}$ can be further expressed as:

(4) $$C_{i}=E_{tx}\left(k,d_{i},n\right)\cdot \left(1+R_{i}\right)+E_{rx}\left(k\right)\cdot R_{i},$$

where

(5) $$d_{i}=\sum_{j=1}^{l}d_{i,j}\cdot x_{i,j},$$

(6) $$R_{i}=\sum_{\begin{array}{c} j=1\\ j\neq i \end{array}}^{l}x_{j,i}.$$

As emphasized in Section 3.1, the lifetime of WBANs is decided by the minimum lifetime of sensor nodes in the network. Therefore, the lifetime of WBANs can be expressed as:

(7) $$Lifetime=\min_{i\in 1,\cdots ,l}\;L_{i}.$$

According to Equations (3)–(7), it can be derived that the lifetime of WBANs is the function of matrix X, which stands for relay selection results:

(8) $$Lifetime\;\left(X\right)=\min_{i\in 1,\cdots ,l}\;L_{i}\left(X\right).$$

As a consequence, aiming to maximize a WBAN lifetime, the optimization problem can be mathematically formulated as:

$$\begin{array}{cc} \underset{X}{\mathrm{maximum}} & Lifetime\;(X),\\ \mathrm{subject}\;\mathrm{to}:\; & \\ \mathrm{Constraints}\;(a):\; & Equations\;(1)-(6)\;and\;(8),\\ \mathrm{Constraints}\;(b):\; & \sum_{j=1}^{l}x_{i,j}=1,\forall i=1,2,\cdots ,l,\\ \mathrm{Constraints}\;(c):\; & x_{i,j}\in \{0,1\},\forall i,j=1,2,\cdots ,l. \end{array}$$

In the optimization problem, constraint (b) stands for the IEEE 802.15.6 topology restriction, while constraint (c) states that each optimization variable is binary. The optimization problem formulated above is an NP-hard binary integer problem, which makes traditional methods difficult to get the optimization results. Therefore, we design a heuristic iterative solution to rapidly address the optimization problem. The solution is introduced in the next section.

## 5. Heuristic Iterative Solution

The fundamental thought of our solution is to iteratively increase the minimum lifetime in the network through adjusting the value of relay selection binary variables in the matrix **X** trying to enhance the network lifetime calculated in Equation (7). The detailed procedure is presented in Algorithm 1. The main steps of the solution in one iteration can be summarized into three major steps:

1. Find the sensor node with the minimum lifetime as the target node to improve its lifetime.
2. Make relay selection action to improve target node’s lifetime according to the condition of the node.
3. Compare the improved minimum lifetime with previous values and decide whether the iteration should be ended or not.

After the iteration has been terminated, the values of each binary variable in the relay selection matrix **X** represent the final relay selection results for the networks.

More specifically, the initialization is to set each node in the WBAN using direct transmission mode and Flag=0, which represents the indicator to terminate the algorithm when it is set to 1. In addition, each node’s lifetime will be calculated for the first time under strategy of all nodes using direct transmission (Line 2–4). Then, the iterative process will begin (Line 6–37): first of all, the target node will be found based on the the value of lifetime (Line 7). Next, different action will be taken depending on whether the target node is a relay node or not, which can be listed as follows:

1. If the target node is a relay node and does not help any other node in cooperative transmission, the algorithm will be terminated due to the fact that the relay node can not use cooperative transmission to save energy. Therefore, there is no possibility for target node to improve its lifetime as well as the whole network (Lines 9–10).
2. If the target node is a relay node and helps some other nodes in cooperative transmission as a relay node, the node with the longest lifetime in the target node’s relayed node list will be removed and set to use direct transmission to reduce the burden of the target node and consequently to increase the lifetime of it (Lines 11–14).
3. If the target node is not a relay node and uses cooperative transmission with the help of a relay node, there will be two conditions:
  (a) If the relay node of the target node is the nearest relay node from the target node, the algorithm will be terminated because it is not possible to select another relay node that can reduce the transmission costs of the target node (Lines 18–19).
  (b) If the relay node of the target node is not the nearest relay node, the nearest relay node will replace current relay node as the target node’s new relay node (Lines 20–23).
4. If the target node is not a relay node and uses direct transmission, the relay node with the longest lifetime will be the new relay node of the target node (Lines 24–27).

**Algorithm 1** Rapid MRS Algorithm
  1:Assume that there are *l* sensor nodes in total in the network including *m* relay nodes.  2:Initialization: Node ID from 1 to *l*, wherein 1 to *m* to relay nodes, $x_{i,i}=1,x_{i,j}=0,$∀i,j=1,2,⋯,l,i≠j, and Flag=0.  3:**for** each i∈[1,l] **do**  4:    Calculate lifetime $L_{i}$, using Equations (3)–(6).  5:**end for**  6:**while** Flag=0 **do**  7:    $n=\underset{i\in [1,l]}{arg\;min_{i}}\left(L_{i}\right)$  8:    **if** n≤m **then**  9:    **if** $R_{n}==0$ **then**10:    Set Flag=1.11:    **else**12:    $k=\underset{x_{i,n}=1}{arg\;max_{i}}\left(L_{i}\right)$13:    Set $x_{k,k}=1,x_{k,i}=0,\forall i\in \left[1,l\right],i\neq k.$14:    **end if**15:    **else**16:    **if** $x_{n,n}$ == 0 **then**17:    Assuming Node ID *r* is the target node’s relay node,18:    **if** $r==\underset{i\in [1,l],i\neq n}{arg\;min_{i}}\left(d_{n,i}\right),$ **then**19:    set Flag=1.20:    **else**21:    $k=\underset{i\in [1,l],i\neq n}{arg\;min_{i}}\left(d_{n,i}\right)$.22:    set $x_{n,k}=1,x_{n,i}=0,\forall i\in \left[1,l\right],i\neq k.$23:    **end if**24:    **else**25:    $k=\underset{i\in [1,m]}{arg\;max_{i}}\left(L_{i}\right)$26:    set $x_{n,k}=1,x_{n,i}=0,\forall i\in \left[1,l\right],i\neq k.$27:    **end if**28:    **end if**29:    **for** each i∈[1,l] **do**30:    Calculate lifetime $L_{i}$, using Equations (3)–(6).31:    **end for**32:    $L_{min}=min\left\{L_{i}|i\in \left[1,l\right]\right\}$33:    Record $L_{min}$ and compare it with previous values.34:    **if** The value of $L_{min}$ does not increase **then**35:    set Flag=1.36:    **end if**37:**end while**


After the relay selection action described above, all the lifetimes in the network will be calculated again and the minimum lifetime among all of the sensor nodes will be recorded and compared with the previous minimum values. If the comparison shows that the minimum value can not increase anymore, the algorithm will come to an end; otherwise, the iteration will continue to process (Lines 29–36).

It should be noticed that the algorithm has a finite number of binary variables and only two variables vary their values in one iteration. Hence, except for the rare conditions described in Lines (9–10 and 18–19), the iteration process described in Algorithm 1 will finally converge to a condition, where certain relay selection binary variables change in a loop mode. The typical value of loop size is two, that is, relay selection results are changed according to the rules specified in Algorithm 1 between two different binary value combinations of **X** in a loop mode (larger values of loop size can also possibly appear in the convergency). The iteration will end when converged to the loop and the binary value combinations of **X** in the loops with larger lifetimes calculated in Equation (7) will be selected as the final results of Algorithm 1. More details on convergency of the proposed rapid solution will be represented in the next section.

## 6. Performance Evaluation

In this section, based on the system model described in Section 3, the performances of LMRSS are evaluated via numerical simulation in terms of network lifetime and time complexity. Moreover, a general WBAN model specified in IEEE 802.15.6 is also employed to comprehensively evaluate the performance of the proposed scheme. In addition to the evaluation, we validate the feasibility of implementation in a real WBAN system for the proposed scheme at the end of this section.

### 6.1. Simulation Setup

We have conducted simulations based on C++ platform. The WBAN in the simulation consists of one coordinator and 16 sensor nodes. Considering that sensor node executing a certain sensing task may not be always deployed at the same position but in a designated area, in order to show generality of sensor node deployment, we classify the human body into six parts in the simulations as shown in Figure 1, which contains head part, main body part, left/right arm part and left/right leg part. The range values of each part are measured from a male human body whose height is 175 cm. In the simulations, we deploy six sensor nodes in main body part and two sensor nodes for every other parts. Sensor nodes are randomly deployed in the parts where they belong. Considering the possible implanted sensors or sensor nodes deployed in the backside of human body, we set the ratio of implanted/back side sensors to the total sensor nodes in the network to 25%.

The node distribution is decided based on the functionality each part contains referring to the general application scenarios for WBAN, where we sort that the main body part is responsible for tissue monitoring sensing like electrocardiogram (ECG) and capsule endoscope, positioning sensing, motion detection and drug delivery, arm parts for blood oxygen and glucose sensors, leg parts for lactic acid sensing, pressure sensing and, if possible, artificial support and head part for electroencephalogram (EEG) sensing. In addition, accelerator nodes can be deployed in all six parts. On the other hand, the main body part is physically more stable than other parts, so, in reality, functional sensor nodes that can be put either in the main body part or other parts are preferred to be deployed in the main body part. As a result, in the simulation scenario, we deploy more sensor nodes in the main body part since its physical stability and the fact that it contains more kinds of functional sensor nodes than other parts. The detailed information for node distribution and body parts are listed in Table 1.

Each sensor node generates a fixed-size packet of *k* bits in one superframe. In the simulation, we set *k* as 1200 bits, which is a typical frame length in WBAN communication according to IEEE 802.15.6 [8]. In addition, we adopt 128 kbps as the transmission rate specified in the IEEE 802.15.6. Depending on this, slot length is set to 10 ms. One superframe contains 65 transmission slots and its duration is set to be 700 ms considering the beacon frame transmission slot, which is in the available range described in IEEE 802.15.6 as well.

We adopt the parameter values in the Nordic nRF2401 low power single chip transceiver (Oslo, Skøyen, Norway), which is widely used in sensor node communication as the energy consumption parameters in Equations (1) and (2). The value of *n* is set to be 3.38 when the channel between transmitter and receiver is LOS (line-of-sight) and 5.9 when channel is NLOS (non-line-of-sight). The values of *n* are adopted from the measurement campaign in [29] and widely used in the WBAN-related literature [21,27,28,30,31]. That is, in Figure 1, the front side sensors get LOS channels with the coordinator while the back side or implanted sensors get NLOS channels with the coordinator. Mean value μ for the energy storage distribution is set to 2 J, and we set a 50% offset from the mean value as the upper and lower bound (Δ=1 J) for the distribution.

When the first sensor node in the WBAN runs out, the simulation stops. The simulation runs for 1000 times, and the averaged results are taken. The system parameters used in the simulation are summarized in Table 2.

### 6.2. Result Analysis

Firstly, before the lifetime performance comparison, the time complexity performance of the heuristic solution and its convergency loop condition are analyzed. From a theoretical perspective, when we use the enumeration method to solve the optimization, the time complexity is $O(2^{n\times n})$. However, when it comes to the proposed heuristic iterative solution, the time complexity is only O(n×n). On the other hand, from the experimental perspective in Table 3, which illustrates the average time cost to solve the optimization in the simulation with the varying number of nodes in the network, the enumeration costs much more than the proposed rapid solution. In more detail, the time taken by enumeration grows very fast with the increasing number of nodes, even more than an hour when the number of nodes is 20 in our simulation. While the proposed solution grows much more smoothly within a hundred microseconds. In a word, the simulation results in Table 3 match the theoretical analysis on time complexity of enumeration and the proposed solution.

**Figure 2:**
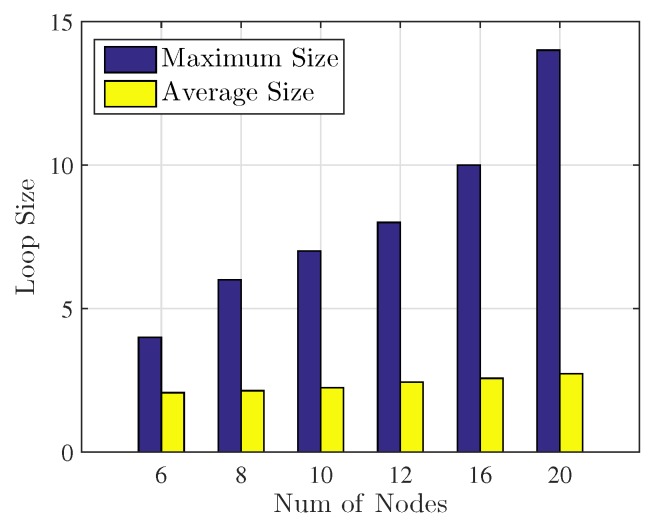
Convergency loop performance comparison.

Figure 2 shows the convergency loop performance of the proposed rapid solution when the iteration ended in the simulation. It can be seen from the figure that the proposed solution always converges to a loop no matter how many nodes are involved (if the iteration can not converge to a loop, then the loop size will be represented by *∞*), which means that the iteration of the proposed solution can always converge to an end in the simulation. The average loop size is close to 2 which indicates that the majority of the loop size in the simulation is 2. Furthermore, it can be seen that when the number of nodes grows, the maximum and average loop size increase due to the fact that the dimensions of **X** become larger, which brings more binary value combinations for the relay selection matrix.

Next, we focus our attention on the performance comparison of network lifetime between our proposed scheme with some existing relay selection schemes: benchmark strategy, where each node in the network only uses direct transmission, the algorithm in [13] (sum-rate algorithm), which is a popular relay selection scheme in WSNs aiming to minimize the total energy consumption, and the WBAN-specific relay selection algorithm in [24] (maxi-rate algorithm), which formulates an optimization problem to minimize the maximum energy consumption rate among sensor nodes. In addition, the optimal value obtained from the optimization using enumeration is also presented in the comparison in order to show the performance gap between the proposed solution and the optimal value.

**Figure 3:**
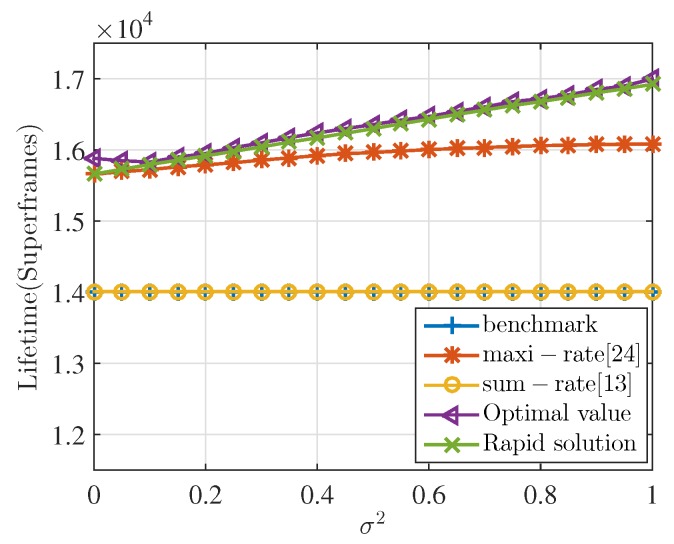
Network lifetime performance comparison.

Figure 3 shows the lifetime performance of each algorithm with the variation of $\sigma^{2}$. The larger the value of $\sigma^{2}$ is, the more serious the energy inequality in the network that exists. The lifetime on the *y*-axis in the unit of superframes represents how many superframes the network gets through before the first sensor node runs out of energy under different relay selection algorithms. That is to say, more superframes means longer lifetime of the network. It can be firstly seen from the figure that the performance of the rapid solution is very close to the optimal value of the optimization. Precisely, the performance gap between the proposed heuristic solution and the optimal value of the optimization is only about 0.5% on average. Combining with the time complexity analysis in Table 3 and Figure 2, it can be inferred that the proposed heuristic iteration described in Algorithm 1 is an effective and time-efficient solution to the optimization problem. In addition, it is confirmed that, throughout the simulations in this paper, performance gap between the rapid solution and the optimal value of the optimization is always very small similar with the results shown in Figure 2. Hence, for simple and clear illustration in the following performance analysis, we will use the results obtained from the rapid solution to represent the performance of LMRSS.

It can be obviously seen from Figure 3 that LMRSS is always the better relay selection scheme in terms of lifetime performance no matter how $\sigma^{2}$ varies compared with the benchmark, sum-rate algorithm and max-rate algorithm. Furthermore, it could be found that with the increasing value of $\sigma^{2}$, the advantages of Rapid MRS over these three algorithms become larger. When $\sigma^{2}=0$, which means that residual energy is all equal to 2 J, the lifetime performance of the proposed algorithm increases by 11.8% compared with the benchmark and sum-rate and gradually goes up to 20.7% when $\sigma^{2}=1$. In addition, the proposed algorithm outperforms the maxi-rate by a gap from 0–7% with the $\sigma^{2}$ variation from 0 to 1. It is because LMRSS considers residual energy conditions of each sensor node together with the energy consumption rate when making relay selection, while sum-rate and maxi-rate only concentrate on energy consumption. As a consequence, the bigger the energy difference degree is, the better lifetime performance the proposed algorithm can get.

In fact, the simulation results indicate that LMRSS always outperforms in the comparison in terms of network lifetime under different numbers of sensor nodes in the body-shaped model. For space restriction, the results are not presented here. However, the same trend of performance deviation under different numbers of nodes is illustrated in a more general WBAN model, which can be referred to Figure 8. It should also be noticed that the sum-rate algorithm is always the same with the benchmark in terms of lifetime, which means that this algorithm loses effectiveness in the environment of WBANs. The reason is that, in WSNs, distances between transmitters and receivers are much larger than WBANs. Using multi-hop transmission can effectively reduce transmission distance, resulting in energy cost decrease. While in WBANs, energy depletion of a sensor node reduced by two-hop transmission is smaller than extra costs for relay nodes. That is to say, when using benchmark strategy, the total consumption of the network is the minimum. As a result, the relay selection scheme obtained from the sum-rate algorithm is consistent with benchmark strategy.

Another observation is that when $\sigma^{2}=0$, the performance of the maxi-rate algorithm is the same with LMRSS and gradually falls behind with the increasing $\sigma^{2}$. This is because when all the sensor nodes have the same amount of energy, minimizing the maximum consumption rate is equivalent to maximizing the minimum lifetime among nodes. When energy difference degree becomes larger, only pursuing to minimize the consumption rate regardless of residual energy of sensor nodes may lead to allocating too much relay burden on a relay node, which relatively lacks residual energy and consequently hinders the enhancement of network lifetime.

**Figure 4:**
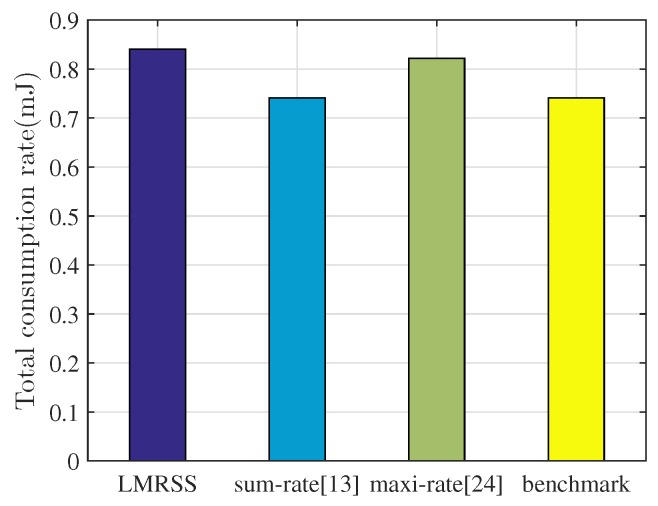
Energy consumption performance comparison.

Figure 4 shows the averaged sum of energy consumption rates of sensor nodes in one superframe under different relay selection algorithms in the simulation. It can be seen that the sum-rate algorithm together with benchmark spends the least because of its optimization objective. LMRSS consumes 13% and 5% more energy than the sum-rate algorithm and maxi-rate algorithm, respectively. It is inferred from the figure that the sum-rate scheme realizes the best energy efficiency, which is the minimum sum of energy consumption rate of all of the sensor nodes in the network, but it does not get the best performance of enhancing network lifetime. Hence, just concentrating on network level energy efficiency is not an appropriate way to prolong network lifetime, as shown in Figure 3. More specifically, in the environment of WBANs, where lifetime may be more important than energy efficiency, LMRSS results in a slight drop in energy efficiency; however, it produces more considerable improvement of network lifetime.

In addition to the explanation for the impact of energy consumption and energy storage of sensor nodes on network lifetime performance, we analyze the worst case for relay selection where sensor nodes in the main body part possess less energy than other nodes in the rest parts. That is, relay nodes are short of energy more seriously than faraway sensor nodes. In this case, LMRSS is out of action since it aims to protect relay nodes that are more energy-restrictive and may run out of energy first. In order to prolong the lifetime of relay nodes, LMRSS will not allocate faraway sensor nodes to any relay nodes so as to cut down on the energy depletion of relay nodes. As a consequence, LMRSS is equal to the direct transmission strategy in this case. On the contrary, the maxi-rate algorithm focuses only on energy consumption of WBANs, and it will still allocate faraway sensor nodes to energy-restrictive relay nodes to achieve its target on energy consumption rate. As a result, relay nodes will accelerate to exhaustion, which, in turn, degrades the network lifetime performance of WBANs. Table 4 shows the network performances of different relay selection methods in the worst case via simulations to validate our analysis above. We set residual energy in sensor nodes belonging to the main body part as 1 J and as 2 J for the other sensor nodes in the network to simulate the worst case in relay selection. As shown in the table, for the maxi-rate algorithm, the maximum consumption rate of sensor nodes within the networks is the lowest. However, its network lifetime performance is decreased by 30% compared with the benchmark strategy since the maxi-rate algorithm only focuses on the energy consumption criterion and neglect energy storage of each sensor node. Meanwhile, as discussed before, LMRSS in this case selects to use direct transmission only, which is the same with benchmark strategy.

**Figure 5:**
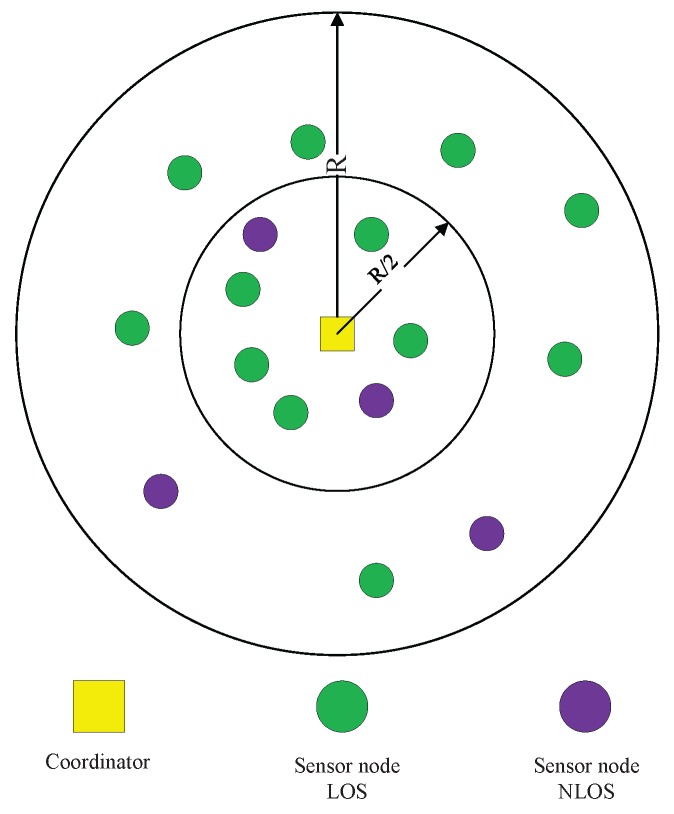
A general WBAN model specified in IEEE 802.15.6.

As stated in Section 1, WBANs are not only specified to be used on the human body. For example, in interactive gaming, robots whose shapes are not restricted to the human form but also tree-form or ball-shaped will also need deploying WBANs to fulfill their mission in the game. Therefore, in this paper, we design a more general WBAN model according to IEEE 802.15.6 to comprehensively evaluate our proposed algorithm. In this standard, the typical network range is regulated to be less than 3 m and the maximum number of nodes in a WBAN is up to 64. The designed model is illustrated in Figure 5. In the figure, the coordinator is placed in the center of a circle whose radius is *R* while sensor nodes are randomly placed in the area of the circle. When the distance of a sensor node from the coordinator is less than R/2, it is treated as a candidate relay node, which, in the figure, is deployed in the circle with the radius of R/2.

**Figure 6:**
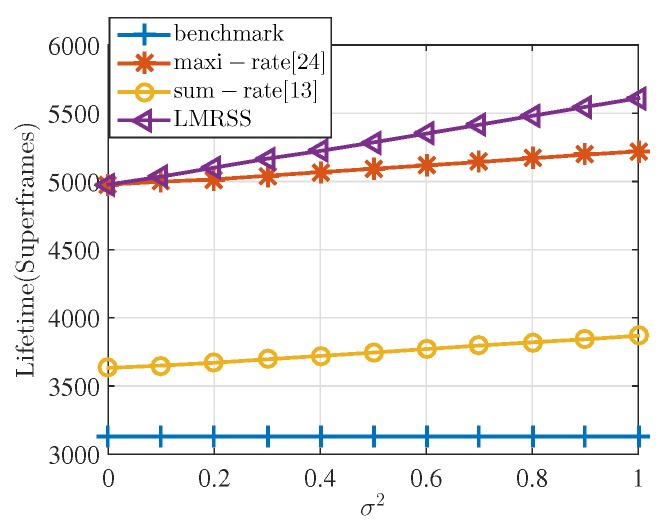
Network lifetime performance comparison on the general WBAN model with the variation of $\sigma^{2}$.

Figure 6 shows the lifetime performance with the variation of $\sigma^{2}$ on the aforementioned WBAN general model. The *R* is set to be 3 m, and the number of nodes is 16 in the simulation. It is illustrated that the proposed algorithm outperforms the other three. In more detail, when $\sigma^{2}=0$, the proposed algorithm improves network lifetime by 59%, 37%, and 0% compared with benchmark, sum-rate algorithm and maxi-rate algorithm, and gradually increases to 79.2%, 45% and 7.4% when $\sigma^{2}$ increases from 0 to 1. The increasing gaps result from the fact that the proposed algorithm considers the residual energy condition of each node as well as the consumption rate. It is also noticed that the performance of the sum-rate algorithm is not the same as the benchmark, which is different from the model in Figure 1. This is because the distance in this model is larger, and two-hop transmission can be selected in certain conditions to effectively reduce the transmission energy for some far-away nodes, resulting in a decrease of the total network energy consumption.

**Figure 7:**
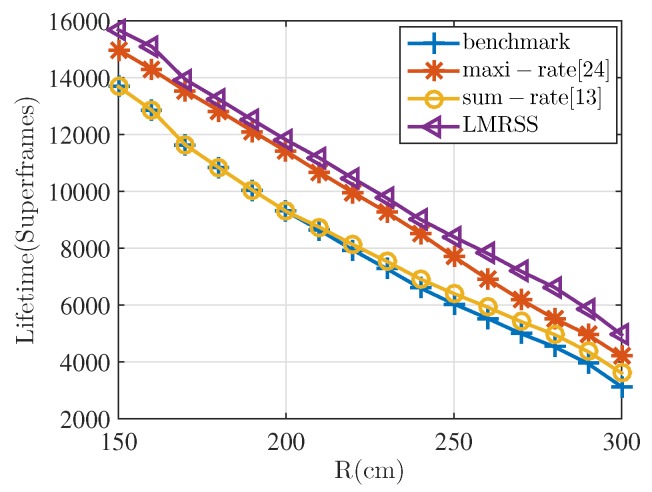
Network lifetime performance comparison on the general WBAN model with the variation of *R*.

Figure 7 shows the lifetime performance with the variation of *R*. The number of nodes is still 16 and the $\sigma^{2}$ is set to be 0.5 in the simulation. It is indicated from the figure that the proposed algorithm still has the best performance no matter how *R* varies. The performance of the sum-rate algorithm is the same as the benchmark when *R* is small and becomes better if *R* is larger than 2 m. LMRSS improves network lifetime by 14% compared with the benchmark and sum-rate algorithm and increases to 59% and 37%, respectively, when *R* is 3 m. Furthermore, when compared with the maxi-rate algorithm, the performance enhancement increases from 4.6% to 18% if the distance varies from 150 cm to 300 cm. It is true from the results that the advantage gap of LMRSS over the other three becomes larger with the increasing value of *R*. The reason for this phenomenon is that with the growing network range, two-hop transmission brings about more transmission distance reduction, which leads to more transmission energy saving. Therefore, the impact of cooperative transmission and its relay selection on network lifetime becomes more and more significant.

**Figure 8:**
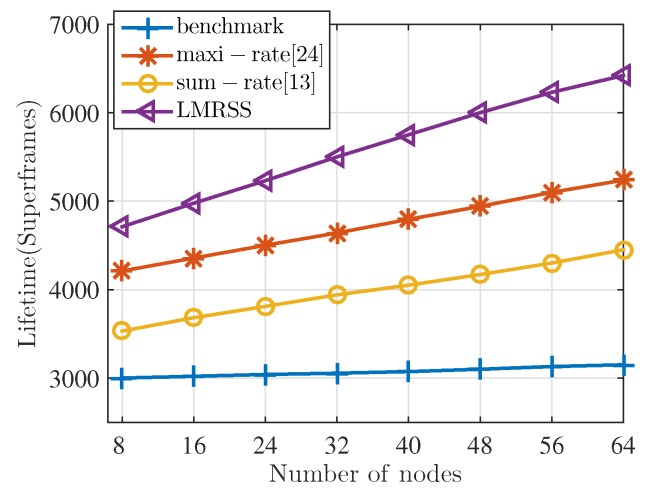
Network lifetime performance comparison on the general WBAN model with a varying number of sensor nodes.

Figure 8 shows the lifetime performance under different total numbers of sensor nodes in WBANs. *R* is set to be 3 m and the $\sigma^{2}$ is set to be 0.5 in this scenario. It can be seen from the figure that, except for the benchmark, which only utilizes direct one-hop transmission to communicate, the other three relay selection schemes will perform better if the total number of sensor nodes increases. The reason for this observation is that more sensor nodes in the network will provide more choices of relay nodes for the sensor nodes, which need two-hop cooperative transmission. As a result, relay selection schemes can allocate more appropriate relay selection to each sensor node in WBANs, which, in turn, prolongs the network lifetime. Furthermore, in the figure, LMRSS outperforms other schemes and the gap between LMRSS and the other two scheme becomes larger with the increasing number of nodes. In particular, the gap between LMRSS and benchmark varies from 57% to 103%, 33.4% to 44.3% for the sum-rate algorithm, and 12% to 22.4% for the maxi-rate algorithm.

To conclude the simulation results presented above, the advantages of LMRSS can be summarized as follows:

- In a body-shaped WBAN application scenario, the proposed scheme can effectively improve the network lifetime of WBANs by 11.8% at least when compared with the direct transmission strategy and is also proved to be better than other existing relay selection methods. A comprehensive comparison between LMRSS, maxi-rate algorithm and sum-rate algorithm is summarized in Table 5 based on the simulation results.
- As LMRSS takes battery capacity diversity into account, it selects relay nodes depending on not only the energy consumption condition but also the remaining energy of each sensor node. When serious energy inequality conditions appear in the network, the advantage of network lifetime improvement of LMRSS is more significant when compared with other schemes.
- The network lifetime advantage of the proposed LMRSS is not restricted in body-shaped WBAN applications. As in a more generalized WBAN model, which is specified in the IEEE 802.15.6 standard, LMRSS still performs better than direct transmission and other existing relay selection methods in terms of network lifetime, no matter how the factors (energy inequality degree, range, number of nodes) vary.
- The time complexity of LMRSS is low, which can be implemented in a real-time WBAN system.

### 6.3. Implementation Discussion

In addition to the performance evaluation of our proposed relay selection scheme through simulations, in this part, we make a brief introduction to how to implement our relay selection scheme in a real WBAN system so as to validate the feasibility of the proposed scheme.

We firstly list the parameters needed to execute our relay selection scheme and describe the methods to obtain the values of these parameters:

- $d_{i,j}$: the distance between each pair of sensor nodes and the distance between sensor nodes and the coordinator;
- *n* : the transmission path state (LOS/NLOS) between each pair of nodes including the coordinator;
- $E_{TXelec},E_{RXelec},E_{amp}$: the energy consumption parameters;
- *k*: the packet length in the network;
- $E_{i}$: the energy storage condition of each sensor node in the network.

As the total number of sensor nodes in a WBAN is not large, $d_{i,j}$ can be measured manually after the sensor nodes have been deployed as well as *n*, e.g., the transmission path state of each pair of nodes according to the location of each node. Furthermore, the energy consumption parameters are obtained depending on the transceiver types of sensor nodes in the networks. *k* is specified by the communication protocol and is easily known by WBAN users. $E_{i}$ can be known by two means depending on two conditions: (A) if the relay selection is invoked at the network initialization where each sensor node is full of energy, we can directly obtain the energy storage information from the sensor node type and battery information; (B) if the relay selection is invoked at a restart from a network recovery, the coordinator can set a request flag in the beacon frame in order to inform each sensor node to report its residual energy condition in the following transmitting time slots. When all of the above parameter values are obtained, the coordinator records this information in its memory and has the ability to execute the relay selection scheme.

Then, we illustrate how to implement our relay selection scheme in two cases. In the first case where a WBAN is at the initialization stage, the coordinator will directly execute the relay selection scheme at the beginning of the first superframe since it has all values needed to execute the relay selection scheme and load the relay allocation results and the corresponding timeslot allocation in the beacon frame. Then, in the beacon transmission slot, the coordinator broadcasts the beacon frame to all of the sensor nodes in the network. As each node must listen and receive this beacon frame, all of the sensor nodes in the network will know their transmission strategy and their relay nodes, if needed.

In the second case, e.g., a WBAN restarts, where the energy storage condition of sensor nodes is unknown, the coordinator sets a request flag in beacon frame at the beginning of the first superframe and allocates timeslots for each sensor node in the network to report their energy storage conditions. As a result, in the first superframe, sensor nodes send their energy storage information to the coordinator in their allocated report slots. After the coordinators have received all of the report frames, it invokes the proposed relay selection scheme and loads the results and the corresponding timeslot allocation in the next beacon frame, which will be broadcasted in the second superframe. Then, in the second superframe, each node will know its transmission strategy and their relay nodes.

It should be emphasized that the low complexity of the rapid solution in our proposed scheme guarantees that the coordinator can finish the algorithm in the interval between the point when the coordinator has received all the energy storage reports to the slot for broadcasting the next beacon frame. Furthermore, it should be noticed that the implementation discussed here does not involve the detailed interaction procedures between the coordinator and sensor nodes that will be further studied in the future.

## 7. Conclusions and Future Work

### 7.1. Conclusions

In this paper, a relay selection scheme is proposed by formulating an optimization problem that considers both the energy consumption rate and residual energy of each node to maximize the minimum lifetime of the nodes in the network. To solve this NP-hard optimization, a heuristic iterative solution is then designed to rapidly address the problem. The simulation results show that the heuristic solution significantly reduces the time complexity with a only 0.5% performance degradation when compared with the optimal values of the optimization, which indicates that the proposed scheme is suitable for a real-time dynamic WBAN system. We evaluate the performance of the proposed scheme on a human-body-shaped model and a general WBAN model specified in the IEEE 802.15.6 standard with respect to energy difference degree, network range and total number of nodes, respectively. It can be illustrated in the simulation results that the proposed scheme always outperforms existing relay selection algorithms in terms of network lifetime of WBANs. In addition, the proposed relay selection algorithm does not violate the topology restriction of IEEE 802.15.6. Furthermore, we make a brief discussion on how to implement our proposed scheme in a real WBAN system to validate the feasibility. In conclusion, LMRSS is an effective and suitable relay selection method to enhance the network lifetime of WBANs.

### 7.2. Future Work

It should be highlighted that the performance evaluation in this paper is only based on the simulation where LMRSS is only invoked once at the initialization (or restart) of the networks. Hence, in the future, we will attempt to investigate the performance of LMRSS in a dynamic fashion where the proposed relay selection scheme will be invoked periodically to make more precious relay selection for WBANs for more lifetime improvement. The feasibility of periodical invoking relies on the simulation results in this paper that the execution time of LMRSS is much smaller than the typical length of one superframe. This time comparison means that LMRSS can be easily invoked in every superframe if wanted. In addition, in order to cooperate with periodical relay selection, the real-time residual energy and location information of each node, which is assumed to be known by the coordinator in this paper, need to be updated by the coordinator (note that the reason for location information updating is WBAN user mobility). Therefore, a method to update the information such as residual energy and location of each node to the coordinator for relay selection needs to be designed next together with the periodical relay selection mechanism.

## Figures and Tables

**Table 1:** Sensor node distribution.

Body Part	Number of Nodes	Range
Head part	2	x∈[−10,10]
		y∈[46,70]
Main body part	6	x∈[−20,20]
		y∈[−25,45]
Left arm part	2	x<−20,y<42
		0.91x+y+38.2>0
Right arm part	2	x>20,y<42
		0.91x−y−38.2<0
Left leg part	2	x∈[−20,−5]
		y∈[−105,−24]
Right leg part	2	x∈[5,20]
		y∈[−105,−24]

The coordinator is at the origin (0,0) of the coordinate graph where right and up are positive directions of *x*-axis and *y*-axis, respectively. The unit in the graph is centimeters.

**Table 2:** Parameter values for simulation.

Parameter	Value
**Network Model**	
Number of sensor nodes *l*	16
Number of relay nodes *m*	6
Packet size *k*	1200 bit
Time slot duration	10 ms
Superframes duration	700 ms
**Energy Consumption Model**	
$E_{TXelec}$	16.7 nJ/bit
$E_{RXelec}$	36.1 nJ/bit
$E_{amp}$	1.97 nJ/bit
μ	2 J
Δ	1 J
n(LOS)	3.38
n(NLOS)	5.9

**Table 3:** Time complexity of the algorithms.

Nodes	Enumeration	The Rapid Solution
6	0.008530s	0.000010s
8	0.084878s	0.000016s
10	0.834952s	0.000017s
12	7.510353s	0.000025s
16	104.487781s	0.000028s
20	6532.097615s	0.000039s

The hardware settings for implementing the simulation in this paper: CPU i-7 2600 3.4 GHz; Memory 8 G RAM.

**Table 4:** Performance in the worst case.

Performance Item	LMRSS	Maxi-Rate [24]	Sum-Rate [13]	Benchmark
Total energy consumption (mJ)	0.7408	0.8218	0.7408	0.7408
Maximum energy consumption (mJ)	0.0814	0.0738	0.0814	0.0814
Network lifetime	14,194.37	9983.49	14,194.37	14,194.37

**Table 5:** Comparison of relay selection algorithms.

Attribute	LMRSS	Maxi-Rate [24]	Sum-Rate [13]
Effectiveness in body-shaped model	Yes	Yes	No
Improvement in body-shaped model(to benchmark)	11.8–20.7%	11.8–14.6%	0%
Effectiveness in general model	Yes	Yes	Only when *R* > 2 m
Improvement in general model (to benchmark)	14–103%	12–67%	0–41%
Time complexity of algorithm	Low	High	Low
Performance in worst case (to benchmark)	No degrading	Degrading	Degrading *

* Sum-rate algorithm will degrade the network lifetime performance when compared with the benchmark in the worst case of the general model if network range is larger than 2 m.
